# Multi-rater developmental trajectories of hyperactivity–impulsivity and inattention symptoms from 1.5 to 17 years: a population-based birth cohort study

**DOI:** 10.1007/s00787-018-1258-1

**Published:** 2018-12-01

**Authors:** Francis Vergunst, Richard E. Tremblay, Cédric Galera, Daniel Nagin, Frank Vitaro, Michel Boivin, Sylvana M. Côté

**Affiliations:** 1 Sainte-Justine University Hospital, Université de Montréal, Montreal, Canada; 2 Department of Pediatrics and Psychology, University of Montreal, Montreal, Canada; 3 School of Public Health, Physiotherapy and Population Science, University College Dublin, Dublin, Ireland; 4 INSERM U1219, Université de Bordeaux, Bordeaux, France; 5 Carnegie Mellon University, Pittsburgh, PA USA; 6 School of Psycho-Education, University of Montreal, Montreal, Canada; 7 School of Psychology, Université Laval, Quebec, Canada; 8 Institute of Genetic, Neurobiological, and Social Foundations of Child Development, Tomsk State University, Tomsk, Russian Federation

**Keywords:** Attention-deficit/hyperactivity disorder, ADHD, Risk factors, Longitudinal

## Abstract

The developmental course of hyperactivity–impulsivity and inattention symptoms from infancy to adolescence has not been documented in a population sample. The aim of this study was to describe the developmental course of hyperactivity–impulsivity and inattention symptoms from 1.5 to 17 years using multiple informants, and to identify perinatal risk factors associated with following elevated (high-risk) trajectories. Using a population-based birth cohort (*n* = 1374), symptom ratings from mothers (1.5–8 years), teachers (6–13 years) and participant self-reports (10–17 years) were combined using group-based multi-trajectory modeling to identify informants’ convergence in identifying high-symptom trajectories of hyperactivity–impulsivity and inattention over time. Perinatal risk factors associated with high-symptom trajectories were identified using stepwise logistic regression. The study found that symptoms of hyperactivity–impulsivity broadly declined from 1.5 to 17 years while symptoms of inattention remained constant. 21.4% of participants followed elevated trajectories of hyperactivity–impulsivity and 20.2% followed elevated trajectories of inattention; 11.6% followed elevated trajectories of both types of symptoms concurrently. Risk factors for high-risk trajectories of hyperactivity–impulsivity were low maternal education, prenatal alcohol exposure, non-intact family, maternal depression, and low child IQ; for high-risk inattention they were prenatal street drug exposure, early motherhood, low maternal education, maternal depression and low child IQ. Risk factors for trajectories of high-risk hyperactivity–impulsivity and inattention concurrently were low maternal education, maternal depression, and low child IQ. The combination of longitudinal assessments from multiple informants (i.e., mother, teacher, participant-reports) provides a new way to characterize hyperactivity–impulsivity and inattention phenotypes over time.

**Electronic supplementary material:**

The online version of this article (10.1007/s00787-018-1258-1) contains supplementary material, which is available to authorized users.

## Introduction

Attention-deficit/hyperactivity disorder (ADHD) is an etiologically complex neurodevelopmental condition associated with significant negative long-term outcomes. Several studies have described the developmental course of hyperactivity–impulsivity and inattention symptoms, but findings remain mixed with respect to symptom continuity and change across childhood and adolescence [1–3]. An important obstacle for studies of this kind is the absence of an accepted methodology that allows a single rater to assess symptoms from infancy through adolescence. However, recent innovations in biostatistics allow us to account for issues of temporality and multi-informant assessments, providing a new way to characterize complex behavioral phenotypes such as ADHD over time.

The present study uses data from a population sample followed from infancy (age 5 months) to adolescence (age 17 years) to identify groups of children following distinct developmental trajectories of hyperactivity–impulsivity and inattention symptoms. Symptom ratings were obtained from three sources—mothers, teachers, and participant-reports—determined by the child’s social and educational contexts (pre-school, elementary school, high school). This approach has several advantages. First, while parents are well placed to describe the children’s behavior in infancy, teachers become the most reliable source of behavioral information once they reach school age [4]. Second, teacher assessments benefit from normative comparisons, something that parental assessments may lack. Third, as children become adolescents, self-rated assessments become a reliable source of behavioral information [5, 6]. Fourth, symptom ratings from multiple informants are important for clinical practice and research as they can help to accurately identify the clinical phenotype of the child [7, 8].

### Early risk factors

Identifying early risk factors for hyperactivity–impulsivity and inattention symptoms is crucial because they constitute the principal pathway through which preventative interventions can be used to reduce risk of onset of ADHD and associated behavioral disorders. Existing studies of early risk factors for ADHD have important limitations [9]. First, most are relatively short and focus on a single developmental period (e.g., early childhood) and consequently fail to account for the developmental nature of the disorder from infancy to adulthood [10]. Second, clinical studies typically rely on discrete diagnostic categories at a single time point, which does not provide information on the developmental nature of symptom change across development [11, 12]. Third, few risk factor studies of hyperactivity–impulsivity and inattention distinguish between the two symptom types, despite evidence that they are best understood as two related but distinct phenotypic dimensions [13, 14]. Fourth, information on risk factors is frequently collected retrospectively, raising the problem of recall bias. Finally, many studies focus on clinical samples or on at-risk males (e.g., pre-term or low birthweight), which limits their generalizability to females and to the general population.

### Aims

The aims of this study were twofold. First, to map the development of hyperactivity–impulsivity and inattention symptoms from infancy to adolescence, and identify groups of children following elevated trajectories, using annual symptom ratings obtained from mothers (early childhood, 1.5–8 years), teachers (middle childhood, 6–13 years) and the participants (adolescence, 10–17 years). Second, to identify early risk factors associated with high-symptom trajectories of hyperactivity–impulsivity, inattention, and both symptom categories concurrently, using multivariable modeling.

## Methods

### Participants and procedure

Data were obtained from the Quebec Longitudinal Study of Child Development (QLSCD) approved by the Quebec Institute of Statistics and the St-Justine Hospital Research Center ethics committees. A population sample of 2120 children born in 1997/1998 in the province of Quebec, were identified through birth registries. Families were included if the pregnancy lasted between 24 and 42 weeks and the mother could speak either French or English. Data were collected through structured interviews conducted by trained researchers. Relevant health and sociodemographic characteristics of the children, family and parents were obtained at 5 months. Behavioral ratings of hyperactivity–impulsivity and inattention were obtained from mother reports (1.5, 2.5, 3.5, 4.5, 5, 6 and 8 years), teacher reports (6, 7, 8, 10, 12 and 13 years), and participant-reports (10, 12, 13, 15 and 17 years). Written informed consent was provided by parents at each interview.

Symptom ratings were derived from items in the early childhood behavior scale from the Canadian National Longitudinal Study of Children and Youth [15]. The instrument incorporates items from the Child Behavior Checklist [16], the Ontario Child Health Study Scales [17], and the Preschool Behavior Questionnaire [18]. Assessments at ages 15 and 17 years were made using the Mental Health and Social Inadaptation Assessment for Adolescents [19]. Hyperactivity–impulsivity items were: Can’t sit still, is restless or hyperactive; Impulsive, acts without thinking; and Difficulty waiting his/her/your turn in games/activities. Inattention items were: Cannot concentrate, cannot pay attention for long; Is inattentive; and Easily distracted, difficulty pursuing any activity. These items correspond with DSM-V criteria for ADHD and correlate highly with those used in other standardized measures of childhood behavioral problems such as the Strengths and Difficulties Questionnaire [20]. These measures have been extensively used in ADHD research as proxies of ADHD diagnosis particularly in epidemiological samples from the general population and consider ADHD as a quantitative trait [9, 21]. Scores were summed and divided by the number of items then standardized on a 0–10 scale. Symptoms were rated on a frequency scale (never/not true = 0, sometimes/somewhat true = 1, often/very true = 2). Alpha scores for hyperactivity–impulsivity and inattention were, respectively, 0.85 and 0.89 for mother ratings (1.5–8 years), 0.91 and 0.93 for teacher ratings (6–13 years), and 0.74 and 0.82 for participant-reports (10–17 years). Correlations for overlapping mother–teacher ratings were, for hyperactivity–impulsivity and inattention, respectively, 0.36 and 0.36 at 6 years and 0.37 and 0.44 at 8 years; correlations for overlapping teacher–participant-reports were 0.24 and 0.38 at 10 years, 0.30 and 0.37 at 12 years, and 0.29 and 0.29 at 13 years.

### Baseline characteristics and early risk factors

Information on family and child characteristics was obtained from parents at 5 months. For categorical variables, the presence of risk was coded as 1 and its absence as 0.

#### Child characteristics

The sex of the child was coded as 1 for boys and 0 for girls. Methylphenidate hydrochloride (Ritalin) use was coded as 1 for any methylphenidate taken between 6 and 15 years (14.4% of the sample). Child IQ was assessed at 41 months using the Wechsler Intelligence Scale for Children Block Design [22]. Child temperament was assessed at age 5 months using the difficult temperament scale from the well-validated Infant Characteristics Questionnaire [23].

#### Prenatal and perinatal factors

Information about the child’s birth was obtained from medical records, defined as: premature birth if < 37th week of gestation (4.9% of children), low birthweight if < 2500 g (3.3% of children). Parental tobacco, alcohol and street drug use during pregnancy were collected when the child was 5 months old. Tobacco, alcohol and drug exposure were, respectively, coded as 1 if the mother smoked at least one cigarette per day (25.3% of mothers), drank at least once per week (3.3% of mothers) or used any drugs (1.4% of mothers) during pregnancy.

#### Perinatal social factors

Family socioeconomic status (SES) was calculated from the family’s overall income, and the mother’s and father’s number of years of education and occupational prestige [24]. SES scores were standardized with a mean of 0 and standard deviation of 1. Family structure was coded as 1 if the family was not intact (i.e., child not living with both biological parents; 21.0% of the sample) and 0 if the family was intact (child living with both their biological parents irrespective of conjugal relationship). Insufficient household income (24.5% of the sample) was calculated based on Statistics Canada’s guidelines which account for family area of residence, number of occupants in the household, and family income over the past year. Early motherhood was coded as 1 if she was 21 years or younger at the birth of her first child (22.5% of the sample). Low parental education was coded as 1 if the mother/father had never obtained a high school diploma (16.0% of mothers, 17.6% of fathers).

#### Postnatal family factors

Family dysfunction at age 5 months was assessed using the McMaster Family Assessment Device [15]. The 12-item instrument measures communication, showing and receiving affection, control of disruptive behavior, and problem resolution. Scores are z-standardized. Mother–child interactions were assessed using the responsiveness scale of the home observation for measurement of the environment–infant version [25]. Hostile–reactive parenting, overprotection, parental self-efficacy, and perceived parental impact were assessed using The Parental Cognition and Conduct Toward the Infant Scale [26]. Scores for each dimension were z-standardized.

#### Parental psychopathology

Parents were asked whether before completing high school they had displayed any of five different conduct problems matching DSM-IV criteria for conduct disorder and antisocial personality disorder. Parental depression, also obtained at 5 months, was assessed using the abbreviated version of the Center for Epidemiologic Studies Depression Scale (12-item) [27]. Parents reported the frequency of depressive symptoms in the past week. Items were coded on a 4-point scale and are z-standardized.

### Trajectory modeling

Ratings of hyperactivity–impulsivity and inattention between 1.5 and 17 years were modeled using group-based multi-trajectory modeling [28, 29]. The method, based on finite mixture modeling, identifies groups of distinctive developmental trajectories over age or time. The approach uses a generalization of the basic trajectory model in which trajectory groups are defined by multiple trajectories. In the present application, each group is defined by trajectories obtained from annual symptoms from three raters: mothers (1.5–8 years), teachers (6–13 years) and participant-reports (10–17 years). The approach generates a set of trajectory groups that represent the continuous symptom course from 1.5 to 17 years. The trajectory groups are displayed separately for each rater (see figures). Model selection was based on methodological as well as substantive considerations. At the methodological level, it was based on the Bayesian Information Criterion (BIC) and Akaike Information Criterion (AIC) numbers and model adequacy tests, while at the substantive level, the model was selected based on parsimony and maximum explanatory power given what is already known about symptom change across development [29]. Further details about model selection, including model fit statistics for the two next best fitting models, are presented in the supplementary material (eTable 1). Separate models were used to estimate hyperactivity–impulsivity and inattention symptoms.

### Multivariable analyses

To test whether individual risk factors significantly distinguished among the six trajectory groups, we ran a series of Wald-based Chi-square tests. Risk factors that were significant at the 0.05 level were then included in a multivariable model to identify risk factors that remained significant in the context of multivariable analysis. Significant predictors were again identified by Wald tests. An important limitation of these tests is that they do not identify which trajectories were distinguished by statistically significant risk factors. From the perspective of developing population-based preventive interventions, we were specifically interested in identifying risk factors for following high-symptom trajectories. In this context, groups of children with atypical (i.e., elevated) symptom levels will be larger than groups of children with the most extreme (i.e., clinical) symptom levels. Thus, to identify children following persistently high symptom trajectories, we combined groups 5 and 6 to create a single high-symptom group and collapsed the remaining four trajectories into a low-symptom group. We then repeated the risk factor analysis within a logistic stepwise regression framework, performed separately for the hyperactivity–impulsivity and inattention symptom categories, then again for those children who were following both high-symptom trajectories simultaneously. To perform this second-stage analysis, participants were assigned to the trajectory group they most likely followed based on the posterior probability of group membership [29], a step that was not required for the analysis of risk factors distinguishing the six trajectory groups.

Three logistic regression models were used to examine early risk factors for high-symptom trajectories: one for inattention, a second for hyperactivity, and a third for participants who followed high-symptom trajectories in both symptom categories simultaneously. In each model, risk factors were identified using two steps. First, we selected variables by running bivariate logistic regressions between each predictor and the outcome (high vs. low trajectory). Variables with *p* values < 0.25 were included in an initial multivariable model (model 1). In the second step, backward selection (variables are deleted if *p* ≥ 0.05) was used together with step-by-step confounding control (model 2) [30]. Results are presented as adjusted odds ratios.

Participants with at least two data points for hyperactivity–impulsivity and inattention for each rater were included in the trajectory modeling (missing data patterns are reported in eTable 2). To examine the effects of missing data on the risk factor analysis, inverse probability weightings were generated (predictors of missingness were sex, insufficient income and maternal depression) and added to the multivariable logistic regression models as a sensitivity analysis. Variables used in the risk factor analysis had between 1.9 and 11.6% missing data. Data were considered missing at random, i.e., missingness is explained by other observed variables [31]. All analyses were conducted using Stata 14. Statistical significance was set at 0.05.

## Results

### Sample characteristics

The initial sample comprised 2120 children. Children with at least two symptom ratings for each rater (mother, teacher, self-reports) were retained for the trajectory analysis (*n* = 1374). Compared with the overall sample, these children were more likely to be male, to come from higher SES households, to have parents who completed high school, and to live in intact families (Table 1).

**Table 1:** Comparison of the study sample with the representative sample

Characteristic^a^	Population sample (*n* = 2120)	Study sample (*n* = 1374)
Sex of the child (male), no (%)	1080 (50.9%)	647^b^ (47.1%)
Lifetime methylphenidate use, no (%)	288 (13.6%)	238^b^ (17.3%)
IQ of the child, mean (SD)	6.20 (3.82)	6.44^b^ (3.88)
Family socioeconomic status, mean (SD)	0.0 (1.0)	0.68^b^ (1.0)
Maternal age at birth of first child (years), mean (SD)	25.8 (4.9)	25.9 (5.0)
Mother high school diploma or higher, mean (SD)	1778 (84.0%)	1175^b^ (85.5%)
Mother no high school diploma, no (%)	339 (16.0%)	198 (14.4%)
Intact family, no (%)	1669 (78.7%)	1099 (80.0%)
Non-intact family, no (%)	443 (20.9%)	272 (19.8%)
Hyperactivity–impulsivity (teacher rated, 8 years), mean (SD)	2.24 (2.63)	2.14 (2.61)
Inattention (teacher rated, 8 years), mean (SD)	3.85 (3.32)	3.72 (3.34)

^a^Between 1.9 and 11.6% missing data

^b^*P* < 0.01

### Developmental trajectories of hyperactivity–impulsivity and inattention

**Fig. 1:**
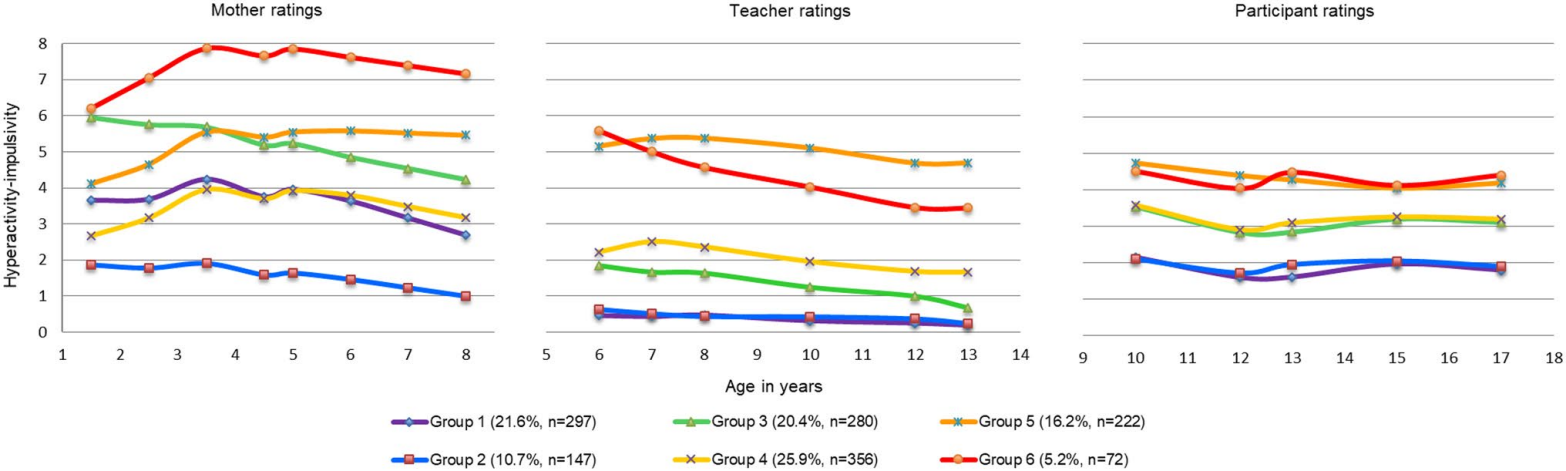
Trajectories of hyperactivity–impulsivity symptoms from 1.5 to 17 years

**Fig. 2:**
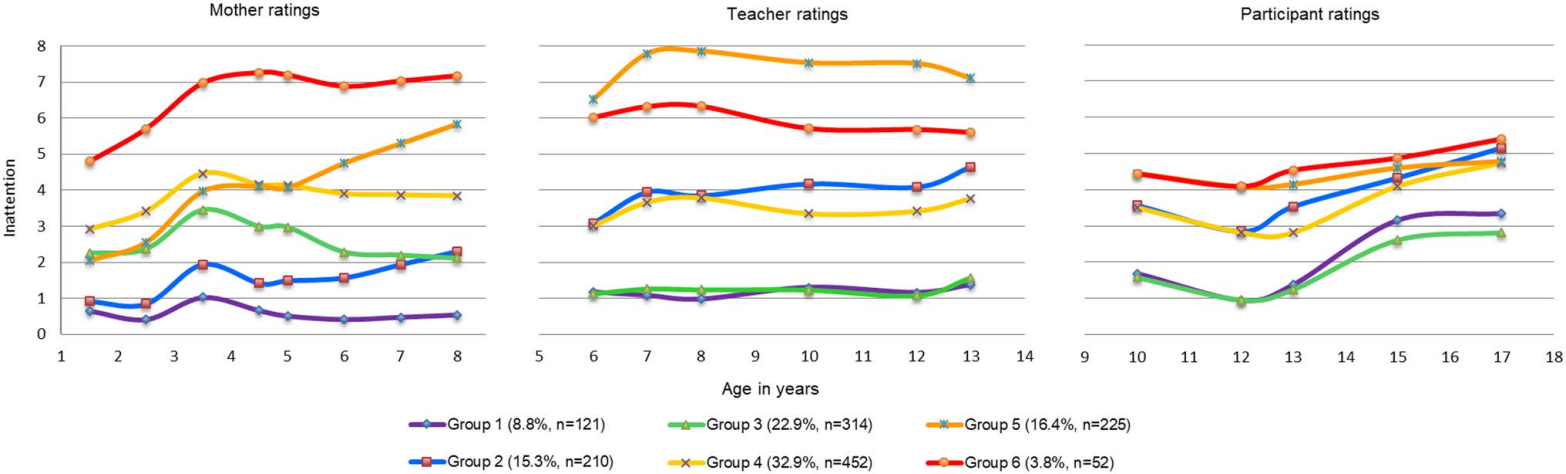
Trajectories of inattention symptoms from 1.5 to 17 years

The developmental trajectories of hyperactivity–impulsivity and inattention symptoms are shown in Figs. 1 and 2. For each symptom category, the overall trajectory of each group is defined by the trajectories from three different raters. For both symptom categories, six-group models provided the best fit of the data based on the Bayesian information criterion [28, 29] and performed well on all tests of model adequacy [29, 32]. Model adequacy tests for the three best fitting models are presented in eTable 1.

A small group of children (5.2%) exhibited persistently elevated hyperactivity–impulsivity symptoms that gradually declined from 1.5 to 17 years. This group, hereafter referred to as “chronic declining”, exhibited high symptom levels from infancy into early childhood, declining moderately in middle childhood, then remaining persistently elevated through adolescence. A second group (16.2% of children) also followed a high-symptom trajectory (hereafter referred to as “chronic”) that remained relatively constant from 1.5 to 17 years. In contrast to the chronic declining group, these children began with average symptom levels in early infancy that rose and surpassed the chronic group in middle childhood, then remained elevated through adolescence. Overall, all six hyperactivity–impulsivity symptom trajectories show a general decline from infancy to adolescence, irrespective of the trajectory group and rater.

The inattention symptom trajectories also revealed a chronic declining and chronic group (3.8% and 16.4% of the sample, respectively) following a similar course to children in the hyperactivity–impulsivity symptom trajectories. In contrast to hyperactivity–impulsivity trajectories which generally declined across development, inattention symptoms remained more constant from infancy through adolescence.

Table 2 shows the baseline child, parent and family characteristics associated with trajectory group membership. Compared to children in the low-symptom groups (groups 1–4), children in the high-symptom groups (groups 5 and 6) were more likely to be male, to come from lower SES households, to have parents who did not complete high school, and to live in non-intact families.

**Table 2:** Child, parent and family characteristics associated with trajectories of hyperactivity–impulsivity and inattention symptoms (*n* = 1374)

Characteristic^a^	Hyperactivity–impulsivity			Inattention		
	Low (*n* = 1080)	Chronic (*n* = 222)	Chronic declining (*n* = 72)	Low (*n* = 1097)	Chronic (*n* = 225)	Chronic declining (*n* = 52)
Sex of the child (male), no (%)	431 (39.9%)	170 (76.6%)	46 (63.9%)	459 (41.8%)	152 (59.6%)	36 (69.2%)
Lifetime methylphenidate use, no (%)	115 (10.6%)	90 (40.5%)	33 (45.8%)	85 (7.7%)	124 (48.6%)	29 (55.7%)
IQ of the child, mean (SD)	6.64 (3.93)	6.70 (3.66)	5.67 (3.60)	6.88 (3.95)	4.64 (3.06)	5.0 (3.12)
Difficult temperament	2.36 (1.53)	2.77 (1.54)	3.59 (1.97)	2.44 (1.54)	2.50 (1.65)	2.90 (2.0)
Prenatal and perinatal factors						
Premature birth, no (%)	43 (4.0%)	10 (4.5%)	7 (9.7%)	43 (3.9%)	13 (5.8%)	4 (7.7%)
Low birthweight, no (%)	31 (2.9%)	4 (0.5%)	5 (6.9%)	30 (2.7%)	9 (4.0%)	1 (1.9%)
Prenatal tobacco exposure, no (%)	247 (22.9%)	71 (32.0%)	25 (34.7%)	259 (23.6%)	68 (30.2%)	16 (30.8%)
Prenatal alcohol exposure, no (%)	33 (3.1%)	9 (4.1%)	5 (6.9%)	35 (3.2%)	10 (4.4%)	2 (3.9%)
Prenatal street drug exposure, no (%)	10 (0.9%)	9 (4.1%)	2 (2.8%)	12 (1.1%)	5 (2.2%)	4 (7.7%)
Perinatal social factors						
Non-intact family, no (%)	193 (17.9%)	59 (26.6%)	20 (27.7%)	200 (18.2%)	59 (26.2%)	13 (25.0%)
Insufficient income, no (%)	210 (19.4%)	61 (27.5%)	10 (13.9%)	206 (18.8%)	68 (30.5%)	17 (32.7%)
Early motherhood (≤ 21-years), no (%)	196 (18.1%)	73 (32.9%)	23 (31.9%)	198 (18.0%)	73 (32.4%)	21 (40.38%)
Low maternal education, no (%)	130 (12.0%)	52 (23.4%)	16 (22.2%)	137 (12.5%)	52 (23.1%)	9 (17.3%)
Low paternal education, no (%)	153 (14.2%)	45 (20.3%)	15 (20.8%)	160 (14.6%)	42 (18.7%)	11 (21.2%)
Postnatal family factors						
Family dysfunction, mean (SD)	1.65 (1.40)	1.80 (1.53)	1.82 (1.42)	1.65 (1.44)	1.82 (1.31)	1.76 (1.39)
Hostile–reactive parenting, mean (SD)	1.0 (1.17)	1.20 (1.22)	1.32 (1.36)	1.05 (1.18)	1.06 (1.17)	1.39 (1.40)
Over protection, mean (SD)	4.52 (2.21)	4.50 (2.19)	5.02 (2.34)	4.49 (2.22)	4.81 (2.16)	4.61 (2.41)
Consequential parenting, mean (SD)	6.96 (1.38)	6.85 (1.37)	6.67 (1.39)	6.96 (1.37)	6.82 (1.37)	6.64 (1.64)
Parental self-efficacy, mean (SD)	8.99 (0.96)	8.97 (0.89)	8.86 (1.0)	8.97 (0.95)	9.06 (0.93)	8.83 (1.07)
Perceived parental impact, mean (SD)	8.52 (1.71)	8.41 (1.76)	7.76 (2.23)	8.51 (1.70)	8.29 (1.95)	8.26 (1.97)
Mother–child interaction, mean (SD)	9.67 (0.73)	9.71 (0.58)	9.73 (0.69)	9.67 (0.72)	9.78 (0.51)	9.56 (0.97)
Parental psychopathology						
Maternal adolescent antisocial behavior, mean (SD)	0.75 (0.88)	1.01 (1.0)	1.06 (1.07)	0.78 (0.91)	0.93 (0.94)	0.83 (0.95)
Paternal adolescent antisocial behavior, mean (SD)	0.65 (0.92)	0.82 (1.02)	0.86 (0.82)	0.65 (0.91)	0.82 (1.05)	0.91 (0.96)
Maternal depression, mean (SD)	1.27 (1.21)	1.68 (1.49)	1.88 (1.56)	1.30 (1.28)	1.48 (1.23)	2.25 (1.60)
Paternal depression, mean (SD)	0.99 (0.95)	1.04 (1.91)	1.20 (1.29)	0.98 (0.94)	1.06 (0.93)	1.39 (1.37)

^a^Between 1.9 and 11.6% missing data

### Early risk factors for high hyperactivity–impulsivity and inattention symptom trajectories

The Wald test comparison of risk factors that significantly distinguished among the six trajectories of hyperactivity–impulsivity in the multivariable model were: sex (*Χ*^2^ = 83.8, *p* = 0.001), child IQ *Χ*^2^ = 14.13, *p* = 0.015), difficult temperament (*Χ*^2^ = 58.28, *p* = 0.001), and maternal depression (*Χ*^2^ = 17.30, *p* = 0.004); for inattention they were: sex (*Χ*^2^ = 71.72, *p* = 0.001), child IQ (*Χ*^2^ = 58.17, *p* = 0.001), difficult temperament (*Χ*^2^ = 15.72, *p* = 0.007), paternal antisocial behavior (*Χ*^2^ = 14.03, *p* = 0.015), and maternal depression (*Χ*^2^ = 12.13, *p* = 0.033).

To create a single high-symptom group in each symptom category, we combined groups 5 and 6 for hyperactivity–impulsivity (*n* = 294, 21.4% of the sample) and did the same for inattention (*n* = 277, 20.2% of the sample), which were then compared with the remaining groups (1 to 4) in the respective symptom categories. Table 3 shows the regression models testing the association between early risk factors and high-symptom trajectories of hyperactivity–impulsivity and inattention. Risk factors associated with the high-symptom trajectories in both symptom categories concurrently (*n* = 160, 11.6%) are shown in the final column.

**Table 3:** Multiple logistic regression models predicting high-symptom trajectories of hyperactivity–impulsivity and inattention

Predictors	Hyperactivity–impulsivity		Inattention		Hyperactivity–impulsivity and inattention	
	Adjusted OR (95% CI)		Adjusted OR (95% CI)		Adjusted OR (95% CI)	
	Model 1	Model 2	Model 1	Model 2	Model 1	Model 2
Sex of the child (male), no (%)	**4.32 (3.04–6.15)**	**4.32 (3.05–6.10)**	**2.56 (1.82–3.60)**	**2.66 (1.90–3.72)**	**4.63 (2.85–7.52)**	**4.78 (2.97–7.69)**
IQ of the child, mean (SD)	**0.93 (0.89–0.98)**	**0.93 (0.89–0.98)**	1.0 (0.90–1.11)	**0.84 (0.80–0.86)**	1.06 (0.93–1.21)	**0.86 (0.80–0.92)**
Difficult temperament, mean (SD)	**1.19 (1.08–1.32)**	**1.21 (1.09–1.33)**	0.83 (0.79–0.88)		0.85 (0.79–0.91)	
Prenatal and perinatal factors						
Premature birth, no (%)	1.64 (0.78–3.48)		1.34 (0.61–2.93)			
Low birthweight, no (%)						
Prenatal tobacco exposure, no (%)	1.21 (0.80–1.81)		0.71 (0.46–1.09)		0.78 (0.45–1.33)	
Prenatal alcohol exposure, no (%)	**2.60 (1.18–5.72)**	**2.49 (1.67–5.33)**	1.42 (0.59–3.39)		2.38 (0.88–6.39)	
Prenatal street drug exposure, no (%)	1.36 (0.35–5.38)		**3.87 (1.04–14.45)**	**3.80 (1.08–13.35)**	2.06 (0.43–9.87)	
Perinatal social factors						
Non-intact family, no (%)	1.42 (0.89–2.23)	**1.55 (1.01–2.39)**	1.0 (0.63–1.60)		1.45 (0.83–2.54)	
Insufficient income, no (%)	0.64 (0.40–1.05)		1.0 (0.63–1.57)		0.53 (0.29–0.98)	
Early motherhood (≤ 21-years), no (%)	1.28 (0.82–1.99)		**1.73 (1.12–3.66)**	**1.69 (1.14–2.52)**	1.49 (0.88–5.39)	
Low maternal education, no (%)	**2.05 (1.22–3.42)**	**2.34 (1.49–3.68)**	1.89 (1.14–3.15)	**1.69 (1.05–2.72)**	**2.97 (1.63–5.39)**	**3.04 (1.18–5.06)**
Low paternal education, no (%)	1.09 (0.67–1.76)		0.87 (0.54–1.41)		1.21 (0.68–2.18)	
Postnatal family factors						
Family dysfunction, mean (SD)	0.90 (0.79–1.04)		1.0 (0.87–1.35)			
Hostile–reactive parenting, mean (SD)	1.04 (0.90–1.21)				0.98 (0.82–1.18)	
Over protection, mean (SD)	1.02 (0.94–1.19)				0.99 (0.89–1.10)	
Consequential parenting, mean (SD)	0.97 (0.86–1.10)		0.99 (0.87–1.11)		0.98 (0.84–1.44)	
Parental self-efficacy, mean (SD)						
Perceived parental impact, mean (SD)	0.93 (0.81–1.05)		0.99 (0.89–1.10)		0.97 (0.85–1.10)	
Mother–child interaction, mean (SD)			1.19 (0.91–1.54)			
Parental psychopathology						
Maternal adolescent antisocial behavior, mean (SD)	1.10 (0.91–1.32)		1.01 (0.84–1.22)			
Paternal adolescent antisocial behavior, mean (SD)	1.09 (0.91–1.29)		1.18 (0.99–1.40)		1.08 (0.87–1.34)	
Maternal depression, mean (SD)	**1.25 (1.09–1.44)**	**1.24 (1.10–1.41)**	**1.19 (1.03–1.38)**	**1.19 (1.05–1.35)**	**1.31 (1.11–1.56)**	**1.31 (1.14–1.53)**
Paternal depression, mean (SD)			1.11 (0.92–1.33)		1.04 (0.82–1.32)	

Predictors significant at the 0.05 level are indicated in bold

*OR* odds ratio

The multivariable logistic regression models in which all risk factors associated with *p* values of < 0.25 in the initial screening (model 1) were significant for both hyperactivity–impulsivity (Wald *Χ*^2^ = 127.78, *p* = 0.0001) and inattention (Wald *Χ*^2^ = 110.04, *p* = 0.0001) and for both models the fit was good: *p* = 0.63 and *p* = 0.82, respectively. For the multivariable analysis with backward selection and step-by-step confounding control (model 2) the models were significant for hyperactivity–impulsivity (Wald *Χ*^2^ = 35.58, *p* = 0.0001) and inattention (Wald *Χ*^2^ = 46.70, *p* = 0.0001), and the fit was good: *p* = 0.75 and *p* = 0.77, respectively. For the analysis of risk factors associated with the high-symptom trajectories for both symptom categories, models 1 and 2 were significant (Wald *Χ*^2^ = 93.80, *p* = 0.0001; Wald *Χ*^2^ = 30.29, *p* = 0.0001) and the fit for both was good (*p* = 0.51 and *p* = 0.34, respectively).

Male sex, prenatal alcohol exposure, low maternal education, non-intact family, maternal depression, difficult temperament, and low child IQ were associated with high hyperactivity–impulsivity symptom trajectories from 1.5 to 17 years. Male sex, prenatal street drug exposure, early motherhood, low maternal education, maternal depression, and low child IQ were significantly associated with high inattention symptom trajectories. For children following high trajectories in both symptom categories, male sex, low maternal education, maternal depression, and low child IQ were significant in the final model. To examine effects of missing data, the multivariable regression models were re-run after including the inverse probability weightings. This did not alter the significance of the results.

## Discussion

This is the first study to describe the course of hyperactivity–impulsivity and inattention symptoms from 1.5 to 17 years in a representative population sample [10], to generate symptom trajectories using an innovative multi-informant approach, and to identify a range of risk factors independently associated with high-symptom trajectories from infancy to adolescence.

By combining symptom ratings from three informants over three distinct developmental periods—mother ratings for early childhood, teacher rating for middle childhood and the participants themselves during adolescence—we provide one of the longest follow-ups of hyperactivity–impulsivity and inattention symptoms published to date. Across the three rater groups, high-symptom trajectories were identified for both symptom types indicating concordance between raters on the children presenting with the highest symptom levels.

### Trajectories of hyperactivity–impulsivity and inattention symptoms

Two groups of children followed high-symptom trajectories of hyperactivity–impulsivity. A chronic declining group, comprising 5.2% of the sample, followed a persistently high trajectory, while a second, comprising 16.2% of the sample, followed a chronic trajectory. There was a general decline in symptoms across development and across raters. This is consistent with reports in the literature showing that hyperactivity–impulsivity symptoms generally decrease from infancy into adulthood [1–3, 33–36].

For inattention, two groups of children followed high-symptom trajectories: 3.8% on a chronic declining trajectory and 16.4% on a chronic trajectory. The overall trend of symptom levels across development was more constant than for hyperactivity–impulsivity. Previous studies of inattention symptoms are mixed with respect to the course of inattention symptoms across development: some show symptoms decline from infancy to early childhood [2, 3, 33, 36] and from middle childhood to adulthood [1], others indicate symptoms remain constant in middle childhood (7–12 years) [34], and yet others suggest they may increase from middle childhood to adolescence [37]. Our data show that following an increase between 1.5 and 3.5 years, inattention symptom remain steady from early childhood to late adolescence irrespective of rater type.

The trajectory models, when compared across the three raters, reveal several distinct features. First, there is more variation in the spread of trajectory groups derived from mother than teacher or self-report ratings. This heterogeneity could reduce overlap in scores between the raters. However, the purpose of the present study was to identify groups of individuals whose trajectories for one or more of the raters but not necessarily for all differed from the others. In this regard, it is still substantively interesting if a group were to differ for only one of the raters. Second, there is a conspicuous rise in children’s self-reported symptoms ratings in early adolescence. This symptom pick-up could reflect an actual increase in symptoms associated with entering middle adolescence, or, though speculative, changes in the child’s subjective perception of their own behavior, possibly in relation to changing peer norms or increasing academic expectations and school pressure.

### Early risk factors for hyperactivity–impulsivity and inattention symptoms

Among children in this study, 21.4% followed high-symptom trajectories of hyperactivity–impulsivity across raters, 20.2% high-symptom trajectories of inattention across raters, and 11.6% followed both concurrently. Risk factors that distinguished among the six trajectory groups based on the initial Wald tests were similar to those identified in the final logistic stepwise regression models. These risk factors are consistent with those reported in several previous studies [9, 38–40]. The risk factors most consistently linked to following high-symptom trajectories of hyperactivity–impulsivity and inattention, as well as both simultaneously, were maternal depression and low maternal education. There is now ample evidence supporting a link between pre and postnatal depression in mothers and ADHD symptoms in offspring [41], but more work is needed to unpick the psychosocial and biological—particularly genetic—contributions which are known to interact in complex ways to increase risk [42]. Low maternal education has also been linked to increased risk of ADHD, but mechanisms underlying this association remain uncertain [43].

### Strengths and limitations

This study provides a novel approach for combining symptom ratings from multiple sources, is one of the longest follow-ups of hyperactivity-impulsivity and inattention symptoms conducted to date, and the first to examine such a wide range of perinatal risk factors associated with following high-symptom trajectories over this extended period. However, the study has several limitations. Attrition is common limitation of long-term follow-up studies including this one. The loss of children from lower SES backgrounds could reduce the generalizability of the study findings. This study does not consider late onset of hyperactivity and inattention symptoms, so the effect of these un-measured putative late-onset cases on the symptom trajectories is unknown. This should be examined in future studies. Finally, this study did not account for genetic factors and genetic confounding (e.g., gene–environment correlation) cannot be ruled out. Recent evidence suggests that genetic factors may interact with exposure to pre- and perinatal risk factors for ADHD with effects decreasing over time [44]; this suggests that age may also need to be taken into account when examining risk factors. Future work on early risk factors should employ genetically informed designs, where possible, and consider the effects of age.

There is an ongoing debate within the ADHD literature concerning the use of symptom ratings obtained from multiple informants. Much of the discussion focuses on the issue of low agreement between informants (e.g., parents and adolescents) which is highly relevant in the context of clinical diagnosis. While this issue warrants further methodological work, the present study was not concerned with the symptom course of ADHD per se, or the risk factors associated with a formal diagnosis of the disorder; rather, the aim was to describe the developmental course of hyperactivity–impulsivity and inattention symptoms from infancy to adolescence in a population sample, based on the perspective of the available raters at each of these developmental periods. We suggest that the use of repeated ‘snapshots’ of symptoms over time, with overlapping assessments in middle and late childhood, may help to more accurately capture and characterize the developmental course of the hyperactivity–impulsivity and inattention phenotypes [7, 8].

A further challenge raised by the multi-rater approach concerns whether symptom ratings made by different raters are equally valid. Evidence from several recent studies suggests that the predictive validity of adolescent self-reports is lower than that of parent reports—at least in certain contexts such as predicting longer-term life outcomes. This has led to the suggestion that parent reports should be favored over self-reports where possible [45, 46]. One explanation for this observation is that children underestimate the severity of their own symptoms [47]. If correct, this finding implies that the self-rated trajectories presented in this study may be a conservative estimate of symptoms compared to what would be observed in the real world.

Resolving differences in symptom ratings across multiple informants is a longstanding problem in developmental psychology and psychopathology and we do not purport to settle the matter with the use of multi-trajectory modeling. We do, however, view the methodology as a valuable methodological device for combining the ratings across raters in a way that transparently highlights commonalities and differences in symptom ratings over time.

## Conclusions

This study found that approximately one-fifth of children follow relatively high symptom trajectories of hyperactivity–impulsivity and inattention, with roughly 11% following elevated trajectories in both symptom categories simultaneously. Hyperactivity–impulsivity symptoms broadly declined from 1.5 to 17 years while inattention symptoms remained constant. A range of perinatal risk factors were associated with following high-symptom trajectories from infancy to adolescence. The study presents a new approach for combining ratings from multiple sources to describe symptom continuity and change over time.

## Electronic supplementary material

Below is the link to the electronic supplementary material.

Supplementary material 1 (DOCX 33 kb)

## References

[1] Döpfner M, Hautmann C, Görtz-Dorten A (2015). Long-term course of ADHD symptoms from childhood to early adulthood in a community sample. Eur Child Adolesc Psychiatry.

[2] Evans SW, Brady CE, Harrison JR (2013). Measuring ADHD and ODD symptoms and impairment using high school teachers’ ratings. J Clin Child Adolesc Psychol.

[3] Musser ED, Karalunas SL, Dieckmann N (2016). Attention-deficit/hyperactivity disorder developmental trajectories related to parental expressed emotion. J Abnorm Psychol.

[4] DuPaul GJ (1991). Parent and teacher ratings of ADHD symptoms: psychometric properties in a community-based sample. J Clin Child Psychol.

[5] Schaughency E, McGee R, Raja SN (1994). Self-reported inattention, impulsivity, and hyperactivity at ages 15 and 18 years in the general population. J Am Acad Child Adolesc Psychiatry.

[6] Ustun B, Adler LA, Rudin C (2017). The World Health Organization adult attention-deficit/Hyperactivity Disorder Self-Report Screening Scale for DSM-5. JAMA Psychiatry.

[7] Martel MM, Markon K, Smith GT (2017). Research review: multi-informant integration in child and adolescent psychopathology diagnosis. J Child Psychol Psychiatry.

[8] APA (2013). Diagnostic and statistical manual of mental disorders.

[9] Galéra C, Côté SM, Bouvard MP (2011). Early risk factors for hyperactivity–impulsivity and inattention trajectories from age 17 months to 8 years. Arch Gen Psychiatry.

[10] Cherkasova M, Sulla EM, Dalena KL (2013). Developmental course of attention deficit hyperactivity disorder and its predictors. J Can Acad Child Adolesc Psychiatry.

[11] Snowling M (2009). Editorial: multiple perspectives on ADHD: implications for future research. J Child Psychol Psychiatry.

[12] Polanczyk G, de Lima MS, Horta BL (2007). The worldwide prevalence of ADHD: a systematic review and metaregression analysis. Am J Psychiatry.

[13] Sonuga-Barke EJS (2010). Editorial: “It’s the environment stupid!” on epigenetics, programming and plasticity in child mental health. J Child Psychol Psychiatry.

[14] Frazier TW, Youngstrom EA, Naugle RI (2007). The latent structure of attention-deficit/hyperactivity disorder in a clinic-referred sample. Neuropsychology.

[15] Statistics Canada (1995). Overview of survey instruments for 1994–1995 data collection, cycle 1.

[16] Achenbach TM, Edelbrock C (1991) Child behavior checklist. Department of Psychiatry, University of Vermont, Burlington, Vermont. https://scholar.google.com/citations?user=gfIVlCIAAAAJ&hl=en

[17] Boyle MH, Offord DR, Racine Y (1993). Evaluation of the original Ontario Child Health Study scales. Can J Psychiatry Rev Can Psychiatr.

[18] Tremblay RE, Desmarais-Gervais L, Gagnon C, Charlebois P (1987). The Preschool Behaviour Questionnaire: stability of its factor structure between cultures, sexes, ages and socioeconomic classes. Int J Behav Dev.

[19] Côté SM, Orri M, Brendgen M (2017). Psychometric properties of the mental health and social inadaptation assessment for adolescents (MIA) in a population-based sample. Int J Methods Psychiatr Res.

[20] Goodman R, Scott S (1999). Comparing the Strengths and Difficulties Questionnaire and the child behavior checklist: is small beautiful?. J Abnorm Child Psychol.

[21] Romano E, Tremblay RE, Farhat A, Côté S (2006). Development and prediction of hyperactive symptoms from 2 to 7 years in a population-based sample. Pediatrics.

[22] Wechsler D (1991). Wechsler Intelligence Scale for children.

[23] Bates JE, Freeland CA, Lounsbury ML (1979). Measurement of infant difficultness. Child Dev.

[24] Willms D, Shields M (1996). A measure of socioeconomic status for the National Longitudinal Study of Children.

[25] Bradley RH, Caldwell BM (1984). The relation of infants’ home environments to achievement test performance in first grade: a follow-up study. Child Dev.

[26] Boivin M, Pérusse D, Dionne G (2005). The genetic-environmental etiology of parents’ perceptions and self-assessed behaviours toward their 5-month-old infants in a large twin and singleton sample. J Child Psychol Psychiatry.

[27] Radloff LS (1977). The CES-D Scale: a Self-Report Depression Scale for research in the general population. Appl Psychol Meas.

[28] Nagin DS, Jones BL, Lima Passos V, Tremblay RE (2016). Group-based multi-trajectory modeling. Stat Methods Med Res.

[29] Nagin D (2005). Group-based modeling of development.

[30] Hosmer DW, Lemeshow S, Sturdivant RX (2013). Applied logistic regression.

[31] Bhaskaran K, Smeeth L (2014). What is the difference between missing completely at random and missing at random?. Int J Epidemiol.

[32] Klijn SL, Weijenberg MP, Lemmens P (2015). Introducing the fit-criteria assessment plot—A Visualisation tool to assist class enumeration in group-based trajectory modelling. Stat Methods Med Res.

[33] Biederman J, Mick E, Faraone SV (2000). Age-dependent decline of symptoms of attention deficit hyperactivity disorder: impact of remission definition and symptom type. Am J Psychiatry.

[34] Hart EL, Lahey BB, Loeber R (1995). Developmental change in attention-deficit hyperactivity disorder in boys: a four-year longitudinal study. J Abnorm Child Psychol.

[35] Lahey BB, Pelham WE, Loney J (2005). Instability of the DSM-IV Subtypes of ADHD from preschool through elementary school. Arch Gen Psychiatry.

[36] Pingault J-B, Viding E, Galéra C (2015). Genetic and environmental influences on the developmental course of attention-deficit/hyperactivity disorder symptoms from childhood to adolescence. JAMA Psychiatry.

[37] Larsson H, Dilshad R, Lichtenstein P, Barker ED (2011). Developmental trajectories of DSM-IV symptoms of attention-deficit/hyperactivity disorder: genetic effects, family risk and associated psychopathology. J Child Psychol Psychiatry.

[38] Sagiv SK, Epstein JN, Bellinger DC, Korrick SA (2013). Pre- and postnatal risk factors for ADHD in a nonclinical pediatric population. J Atten Disord.

[39] Froehlich TE, Lanphear BP, Auinger P (2009). Association of tobacco and lead exposures with attention-deficit/hyperactivity disorder. Pediatrics.

[40] Thapar A, Cooper M, Jefferies R, Stergiakouli E (2012). What causes attention deficit hyperactivity disorder?. Arch Dis Child.

[41] Wolford E, Lahti M, Tuovinen S (2017). Maternal depressive symptoms during and after pregnancy are associated with attention-deficit/hyperactivity disorder symptoms in their 3- to 6-year-old children. PLoS One.

[42] Sfelinioti S, Livaditis M (2017). Association of maternal depression with children’s attention deficit hyperactivity disorder. Psychiatr Psychiatr.

[43] Hjern A, Weitoft GR, Lindblad F (2010). Social adversity predicts ADHD-medication in school children–a national cohort study. Acta Paediatr Oslo Nor.

[44] Brinksma DM, Hoekstra PJ, van den Hoofdakker B (2017). Age-dependent role of pre- and perinatal factors in interaction with genes on ADHD symptoms across adolescence. J Psychiatr Res.

[45] Du Rietz E, Kuja-Halkola R, Brikell I (2017). Predictive validity of parent- and self-rated ADHD symptoms in adolescence on adverse socioeconomic and health outcomes. Eur Child Adolesc Psychiatry.

[46] Vugteveen J, De Bildt A, Hartman CA, Timmerman ME (2018). Using the Dutch multi-informant Strengths and Difficulties Questionnaire (SDQ) to predict adolescent psychiatric diagnoses. Eur Child Adolesc Psychiatry.

[47] Owens JS, Goldfine ME, Evangelista NM (2007). A critical review of self-perceptions and the positive illusory bias in children with ADHD. Clin Child Fam Psychol Rev.

