# Supplementary material for: Multi-rater developmental trajectories of hyperactivity–impulsivity and inattention symptoms from 1.5 to 17 years: a population-based birth cohort study
Source: Eur Child Adolesc Psychiatry. 2018 Dec 1;28(7):973–83. doi: 10.1007/s00787-018-1258-1 (PMC6647515; doi:10.1007/s00787-018-1258-1)
Supplement: Supplementary file 1 — Supplementary material 1 (DOCX 33 kb) [file 787_2018_1258_MOESM1_ESM.docx]

**Supplementary materials**

eTable 1 Participants with complete data at each time point

eFigure 1 BIC numbers and model adequacy tests for 6 group model and two next best fitting models

**eTable 1** Participants with complete data at each time point

|  | **Hyperactivity-impulsivity** | |  | **Inattention** | |
| --- | --- | --- | --- | --- | --- |
|  | **N** | **(%)** |  | **N** | **(%)** |
| **Mother** |  |  |  |  |  |
| 1.5 years | 2038 | (96.1%) |  | 2045 | (96.5%) |
| 2.5 years | 1997 | (94.2%) |  | 1995 | (94.1%) |
| 3.5 years | 1948 | (91.9%) |  | 1949 | (91.9%) |
| 4.5 years | 1942 | (91.6%) |  | 1942 | (91.6%) |
| 5 years | 1759 | (83.0%) |  | 1759 | (83.0%) |
| 6 years | 1492 | (70.4%) |  | 1792 | (70.4%) |
| 8 years | 1450 | (68.4%) |  | 1450 | (68.4%) |
|  |  |  |  |  |  |
| At least 2 data points across all mother ratings | 2012 | (90.5%) |  | 2012 | (90.5%) |
|  |  |  |  |  |  |
| **Teacher** |  |  |  |  |  |
| 6 years | 964 | (45.5%) |  | 966 | (45.6%) |
| 7 years | 1301 | (61.4%) |  | 1303 | (61.5%) |
| 8 years | 1263 | (59.6%) |  | 1282 | (60.5%) |
| 10 years | 983 | (46.4%) |  | 987 | (46.6%) |
| 12 years | 997 | (47.0%) |  | 1004 | (47.4%) |
| 13 years | 945 | (44.6%) |  | 1027 | (48.4%) |
|  |  |  |  |  |  |
| At least 2 data points across all teacher ratings | 1491 | (67.1%) |  | 1496 | (67.3%) |
|  |  |  |  |  |  |
| **Self-reports** |  |  |  |  |  |
| 10 years | 1317 | (62.1%) |  | 1318 | (62.2%) |
| 12 years | 1347 | (63.5%) |  | 1347 | (63.5%) |
| 13 years | 1230 | (58.0%) |  | 1229 | (58.0%) |
| 15 years | 1442 | (68.0%) |  | 1443 | (68.1%) |
| 17 years | 1267 | (59.8%) |  | 1265 | (59.7%) |
|  |  |  |  |  |  |
| At least 2 data points across all self-report ratings | 1518 | (68.3%) |  | 1519 | (68.3%) |

**eTable 2** Model adequacy tests for the 6 group model and two next best fitting models

| **6 group** | **Hyperactivity** | | | |  |  | **Inattention** | | | | |
| --- | --- | --- | --- | --- | --- | --- | --- | --- | --- | --- | --- |
|  | **Count** | **Post Prob. GM** | **OCC** | **Prob obs.** | **Prob**  **expec.** |  | **Count** | **Post Prob. GM** | **OCC** | **Prob obs.** | **Prob**  **expec.** |
| **G1** | 297 | 80.9 | 15.4 | 21.6 | 21.3 |  | 121 | 88.2 | 77.2 | 8.8 | 9.3 |
| **G2** | 147 | 90.7 | 81.5 | 10.7 | 11 |  | 210 | 81.5 | 24.4 | 15.3 | 15.3 |
| **G3** | 280 | 82 | 17.8 | 20.4 | 21 |  | 314 | 84.1 | 17.9 | 22.9 | 22.4 |
| **G4** | 356 | 80.4 | 11.7 | 25.9 | 25.4 |  | 452 | 84.4 | 11 | 32.9 | 32.6 |
| **G5** | 222 | 84.7 | 28.8 | 16.2 | 15.8 |  | 225 | 86.7 | 33.4 | 16.4 | 16.5 |
| **G6** | 72 | 88.9 | 144.3 | 5.2 | 5.6 |  | 52 | 83.7 | 130.9 | 3.8 | 4 |
|  | BIC=-42050.18 (N=21363)  BIC=-41991.19 (N=1374)  AIC=-41878.84  L=-41835.84 | | | | |  | BIC=-44683.17 (N=21466)  BIC=-44624.07 (N=1374)  AIC=-44511.73  L=-44468.73 | | | | |
|  |  | | | | |  |  | | | | |
| **5 group** | **Hyperactivity** | | | | |  | **Inattention** | | | | |
|  | **Count** | **Post Prob. GM** | **OCC** | **Prob obs.** | **Prob**  **expec.** |  | **Count** | **Post**  **Prob.**  **GM** | **OCC** | **Prob obs.** | **Prob**  **expec.** |
| **G1** | 266 | 88.9 | 33.4 | 19.4 | 19.2 |  | 148 | 88.5 | 63.7 | 10.8 | 11.2 |
| **G2** | 300 | 80.2 | 14.5 | 21.8 | 22.8 |  | 379 | 81.1 | 11.3 | 27.6 | 26.9 |
| **G3** | 442 | 84.7 | 11.7 | 32.2 | 31.3 |  | 379 | 85.4 | 15.4 | 27.6 | 27 |
| **G4** | 285 | 85.6 | 22.7 | 20.7 | 20.4 |  | 234 | 82 | 22.3 | 17 | 17.7 |
| **G5** | 81 | 87.3 | 109.7 | 5.9 | 6.2 |  | 234 | 87.4 | 33.9 | 17 | 17.2 |
|  | BIC=-42167.93 (N=21363)  BIC=-42117.17 (N=1374)  AIC=-42020.50  L=-41983.50 | | | | |  | BIC=-44758.68 (N=21466)  BIC=-44707.83 (N=1374)  AIC=-44611.16  L=-44574.16 | | | | |
|  |  | | | | |  |  | | | | |
| **4 group** | **Hyperactivity** | | | | |  | **Inattention** | | | | |
|  | **Count** | **Post Prob.**  **GM** | **OCC** | **Prob obs.** | **Prob**  **expec.** |  | **Count** | **Post Prob.**  **GM** | **OCC** | **Prob obs.** | **Prob**  **expec.** |
| **G1** | 315 | 89.1 | 27.6 | 22.9 | 22.5 |  | 260 | 88.5 | 32.8 | 18.9 | 19.3 |
| **G2** | 432 | 85.2 | 12.6 | 31.4 | 31.3 |  | 209 | 84.9 | 31.2 | 15.2 | 15.9 |
| **G3** | 356 | 82.8 | 13.8 | 25.9 | 26.4 |  | 499 | 85.9 | 10.7 | 36.3 | 35.7 |
| **G4** | 271 | 91.3 | 42.8 | 19.7 | 19.9 |  | 406 | 90.4 | 22.5 | 29.5 | 29.1 |
|  | BIC=-42250.21 (N=21363)  BIC=-42209.05 (N=1374)  AIC=-42130.67  L=-42100.67 | | | | |  | BIC=-44893.63 (N=21466)  BIC=-44841.40 (N=1374)  AIC=-44742.12  L=-44704.12 | | | | |

Post prob. GM = posterior probability of group membership; OCC = odds of correct classification; Prob obs. = probability observed; Prob expec. = probability expected.

The 6-group model was selected based on the Bayesian Information Criterion (BIC) and Akaike Information Criterion (AIC) numbers, model adequacy tests, and the overall explanatory power of the model compared to the next best fitting models (4 and 5 groups). The aim of the study was to identify one or two relatively small groups of children following high-symptom (i.e. high risk) trajectories of hyperactivity and inattention. The 4 and 5 group models did not distinguish these ‘at-risk’ groups rendering them uninformative for purposes of the study. A 7-group model was also considered but was discarded on the principle of parsimony as it provided no information beyond what was already present in the 6-group model. The final 6-group model distinguished two at-risk groups and performed well in terms of fit (larger BIC and AIC numbers) and model adequacy tests. In both models, the average posterior probability of group membership for group assignments based on the maximum posterior probability rule exceeded the .7 threshold recommended by Nagin (2005). Both models also passed the odds of correct classification test suggested by Nagin (2005) and his recommendation that the estimated probability of group membership for each group be close to the proportion assigned to the group.
